# Development of a training programme for primary care mental health staff to support management of depression and anxiety in long-term conditions

**DOI:** 10.1017/S1463423618000658

**Published:** 2018-08-28

**Authors:** Kate Hamilton-Westa, Amanda Batesb, Sarah Hothamc, Patricia Wilsond

**Affiliations:** 1 Reader in Health Psychology, Centre for Health Services Studies, George Allen Wing, University of Kent, Canterbury, Kent, UK; 2 Public Engagement Officer, Centre for Health Services Studies, George Allen Wing, University of Kent, Canterbury, Kent, UK; 3 Research Fellow, Centre for Health Services Studies, George Allen Wing, University of Kent, Canterbury, Kent, UK; 4 Professor of Primary and Community Care, Centre for Health Services Studies, George Allen Wing, University of Kent, Canterbury, Kent, UK

**Keywords:** Chronic obstructive pulmonary disease, Coronary heart disease, Diabetes, Long-term conditions, Mental health, Training

## Abstract

**Aim:**

We aimed to develop, deliver and evaluate a brief training programme for primary care mental health staff in NW London focussing on long-term physical health conditions (LTCs). The objective was to improve participants’ knowledge, understanding and confidence (self-efficacy) in providing effective support to people with LTCs. The second objective was to develop an online version to be made available more widely.

**Background:**

The project was commissioned by NW London Collaboration of Clinical Commissioning Groups as part of a strategy to develop more joined up care and support for people with mental health needs. Training was developed by a team of experts, with input from commissioners, service users, clinicians and service managers.

**Methods:**

Training was delivered via two-day interactive workshops providing: (i) key facts (informed by a review of published research and publically available health information); (ii) opportunity to engage with the ‘lived experience’ of people with LTCs (via videos, role plays, case studies and group discussion); (iii) skills-based training (in specific assessment and intervention methods). Knowledge, understanding and confidence (with respect to supporting people with LTCs) were assessed at the start and end of the training. An online training programme (with embedded evaluation questionnaire) was also developed, covering the same themes as the workshop.

**Findings:**

Mental health staff (*n*=60) reported limited knowledge, understanding and confidence before the workshop, underlining the need for training. Knowledge of LTCs improved significantly following training (*P*<0.0001), along with awareness of the impact of poor psychological wellbeing on physical health (*P*<0.05) and the role of psychological therapies in supporting people with LTCs (*P*<0.0001). Self-efficacy also improved (*P*<0.001). Online training was accessed by 894 participants in the first six months and 187 provided feedback via the evaluation questionnaire. Responses indicated that participants found the training useful (88%), interesting (91%) and easy to understand (97%).

People with long-term physical health conditions (LTCs), also referred to as non-communicable diseases, are at greater risk of poor psychological wellbeing, including diagnosable mental health conditions (Naylor *et al*., 2012) and problems not meeting criteria for formal diagnosis, such as difficulties with stress and coping (NHS Diabetes and Diabetes UK, 2010; NHS Confederation, 2012). People with LTCs find it harder to manage their treatment regimes in the context of reduced psychological wellbeing (NHS Diabetes and Diabetes UK, 2010), and mental health co-morbidities have been linked to poorer clinical outcomes, reduced quality of life and increased health service costs (Naylor *et al*., 2012). Overall morbidity also increased in people with LTCs when mental health problems are present (NHS Confederation, 2012).

In the UK, government policy has highlighted the need to expand mental health service provision to meet the needs of people with LTCs (Department of Health 2011a, b). A competence framework has been developed for carrying out effective clinical work for people with persistent physical health conditions (Roth and Pilling, 2015) and a joint consensus statement of the Royal Colleges of Psychiatrists, general practitioners and physicians. The British Psychological Society (2015) has identified key principles for delivering integrated physical and psychological health care. Evidence-based curricula have been developed to support the commissioning of training for mental health workers, and National Health Service (NHS) England is currently supporting 22 early implementer projects across the country providing support for people with physical health conditions within the framework of the national improving access to psychological therapies (IAPT) programme (https://www.england.nhs.uk/mental-health/adults/iapt/mus/sites/).

At a local level, pressure on services associated with rising prevalence of LTCs and mental health co-morbidities is challenging commissioners to make changes to the way people with mental health needs are supported. Health care organisations are forming new collaborations to respond effectively to local need and develop ‘place-based plans’ (NHS England, 2017a). To improve mental health and wellbeing across NW London, the NW London Collaborative of Clinical Commissioning Groups (CCCG) has established a new strategy called ‘Like minded’, which is about working in partnership to deliver joined-up services that improve the quality of life for individuals, families and communities who experience mental health issues. The ambitions for this work include ensuring that the mental health workforce is able to work effectively with people with LTCs. The current project was commissioned to support this ambition.

## Aim

The main aim of the project was to develop, deliver and evaluate training for primary care mental health staff in NW London focussing on three LTCs, which account for the greatest service spend in the CCCG area: diabetes, chronic obstructive pulmonary disease (COPD) and coronary heart disease (CHD). The objective was to improve participants’ knowledge, understanding and confidence (self-efficacy) in providing effective support to patients with LTCs. The second objective was to develop an online version of the training programme that could be accessed by mental health staff across the NHS.

## Methods

### Participants and procedure

Participants were 60 primary care mental health staff (practitioners delivering talking therapies for depression and anxiety disorders) employed within the eight boroughs of NW London. The group comprised psychological wellbeing practitioners (*n*=41), counsellors (*n*=17), a clinical psychologist (*n*=1) and an assistant psychologist (*n*=1).

Training was delivered (by K.H.W., A.B. and S.H.) as an interactive two-day workshop, with participants offered a choice of three dates during July and August 2017. The number of participants attending each two-day workshop ranged from 19 to 21.

Assessment measures (described in ‘Assessment measures’ section) were completed at the start and end of the training. Responses were anonymous and a unique participant identification number was used to match participants’ baseline and follow-up questionnaires.

### Training content

Training content was developed by a team of experts (in health psychology and primary care) between March and June 2017, with input from stakeholders, including commissioners, service managers, clinicians and service users. Workshops provided: (i) key facts, informed by a review of published research and publically available health information [such as information on prevalence of LTCs, how these are managed (and self-managed) and how they impact psychological wellbeing]; (ii) opportunity to engage with the ‘lived experience’ of people with specific conditions (exploring through videos, role plays, case studies and group discussion what it means to live with and manage chronic illness) and (iii) skills-based training (in the use of specific assessment and intervention methods).

Assessments included the Brief Illness Perceptions Questionnaire (Broadbent *et al*., 2006), Diabetes Distress Scale (Polonsky *et al*., 2005), COPD assessment test (Jones *et al*., 2009) and Minnesota Living with Heart Failure Questionnaire (Rector and Cohn, 1992). Intervention methods are described in the following section.

### Intervention methods

The key principles for supporting patients with long-term conditions were drawn from concordance therapy, which aims to support people to manage their condition skilfully and flexibly, drawing on available sources of support as necessary (Higgins, Livingston and Katona, 2004; Hamilton-West *et al*., 2010; 2013; 2014). The key principles include a non-blaming atmosphere; emphasis on personal choice and responsibility and development of ‘self-efficacy’ (see ‘Theoretical underpinning’ section).

In line with this approach, participants were trained to use open-ended questioning, reflective listening and regular summarising to explore patients’ responses to assessment measures and develop a shared understanding of their individual needs and priorities. This assessment was then used to inform the use of motivational, cognitive-behavioural and educational strategies, including: (i) helping patients to access information about the condition that is relevant to their specific areas of concern and reviewing this with them (links to relevant information and resources were provided in the workshop); (ii) working with the patient to develop ‘SMART goals’ and/or draw up a ‘balance sheet’ to support health behaviour change (the workshop included group exercises to practice using these techniques) and (iii) signposting patients to self-management programmes, the NHS health trainer programme, and/or patient support groups (eg, Diabetes UK, British Lung Foundation, British Heart Foundation) – further information and links were provided.

The Acceptance and Commitment Therapy (ACT) model was also presented, with an emphasis on the importance of supporting patients to live the way they want to live, in accordance with their values (Hayes *et al*., 2006; Gregg *et al*., 2007). Trainers highlighted that the workshop was not designed to teach participants to *deliver* ACT, but to consider: (1) how living with a long-term condition can interfere with valued activities; (2) how patients can be supported to live in accordance with their values in the context of their long-term condition. Participants were shown video interviews of patients discussing their experiences of giving up work due to health problems (accessed via healthtalk.org). They were then asked to reflect on what the patients valued about their work (eg, using their skills/training, feeling ‘useful’, sense of camaraderie/competition) and how they had found other ways to live in accordance with their values after giving up work (eg, by studying, spending time with family, taking up new hobbies and activities).

The importance of delivering therapy within the national IAPT framework, in accordance with relevant guidance^1^ and individual skills and competencies^2^ was emphasised throughout. Participants were reminded that their role is not to provide medical advice and that patients should discuss any concerns about their condition and its management with their doctor/nurse.

### Theoretical underpinning

The approach to training was informed by social cognitive theory (Bandura, 1977), which holds that individuals are more likely to carry out a behaviour and to persist with the behaviour in the face of difficulties if they are high in ‘self-efficacy’. Self-efficacy refers to the individual’s level of confidence in their ability to perform specific behaviours under specific conditions. There are four main routes to developing self-efficacy; mastery experience (ie, performing the behaviour successfully); vicarious experience (ie, observing others performing the behaviour successfully); managing physiological arousal (eg, via discussion of worries and concerns) and supportive feedback.

These four elements were built into the workshop – for example by providing opportunities for participants to practice using specific assessments and intervention methods, to observe others using these methods successfully, express worries and concerns and receive supportive feedback from trainers. Participants were also taught about the concept of self-efficacy and approaches for enhancing patients’ confidence in their ability to perform health-related behaviours.

The approach to training was further informed by Leventhal’s ‘common sense model of illness representations’ (Leventhal *et al*., 1980) – this widely used theory sets out the dimensions along which individuals’ perceptions of illness may vary. It is useful for understanding the individual’s ‘personal model’ of their condition (ie, what the condition and its management means to them) and how this shapes the way each person copes with/manages their condition.

### Online training

**Figure 1:**
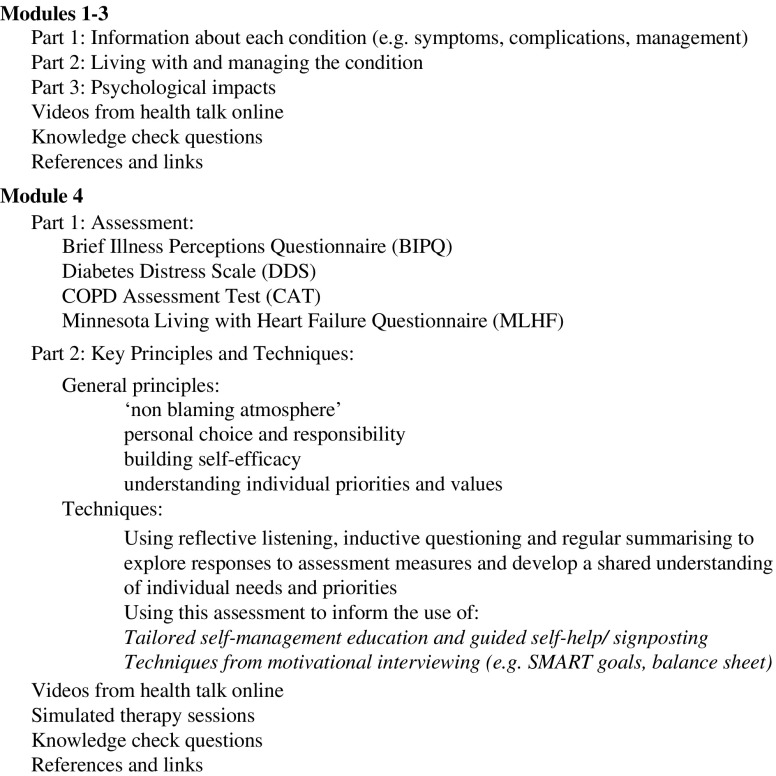
Overview of training module content

An online training programme was developed between July and October 2017, covering the same themes as the workshops, with links to further information and resources. Once the content of training had been reviewed by stakeholders, we worked with a professional web development team to create a fully functioning training resource. This included ‘knowledge check’ questions to self-assess progress against intended learning outcomes and videos of simulated therapy sessions. Stakeholders also reviewed and provided feedback on the structure and format of the online resource and visual content, such as images and logos (http://www.trainingltcs.org.uk/). The resulting resource comprised four modules: the first three focussed on diabetes, COPD and CHD, respectively. Module four focussed on assessment and treatment approaches (see Figure 1).

### Assessment measures

A questionnaire was developed to assess: (i) knowledge and understanding of the LTCs covered in training (10 ‘yes/no’ items, eg, ‘I understand the difference between Type 1 and Type 2 diabetes’); (ii) understanding of relationships between mental and physical health and the role of psychological therapies in supporting people with LTCs (three ‘yes/no’ items, eg, ‘I understand how poor psychological wellbeing can impact physical health in people with LTCs’); (iii) participants’ self-efficacy in relation to using condition-specific assessments and interventions (seven items, scored from 0=‘not at all confident’ to 10=‘very confident’, eg, ‘I am confident that I would be able to select appropriate (condition specific) measures for assessing patients with diabetes, COPD and CHD’); (iv) participants’ experiences of training. Responses to open-ended questions (eg, asking participants to highlight aspects they found useful and ways in which the training could be improved).

A feedback questionnaire embedded in the online training programme asked respondents to indicate whether they found the training useful, interesting and easy to understand (‘yes’, ‘no’ or ‘to some extent’). They were also asked to provide a satisfaction rating from 0 to 10 and invited to comment on what they liked about the training programme and how it could be improved.

### Data analysis

Quantitative data were entered into SPSS (version 22) and both descriptive (means and standard deviations) and inferential statistics (McNemar’s test and paired-samples *t*-tests) were computed. The standard level of significance (*P*<0.05) was used to examine change from baseline to follow-up. Responses to open-ended questions were analysed using a content analytic approach.

For the online training, feedback data were downloaded six months after the programme launched. Data were analysed to examine uptake of the training programme (number of participants accessing the programme in the first month) as well as user satisfaction and suggestions for improvement.

## Results

### Training workshops

**Figure 2:**
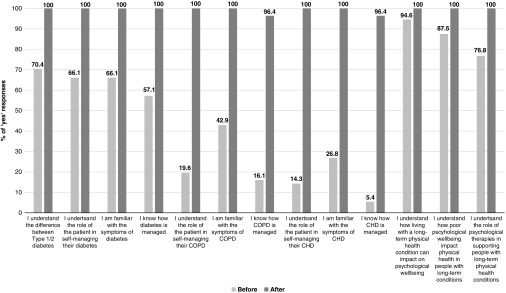
Knowledge and understanding scores at baseline and follow-up

Completed questionnaires were received from 56 participants (93.3%) at the start and 55 participants (91.7%) at the end of training. The proportion of ‘yes’ responses (indicating agreement with the knowledge and understanding statements) at baseline and follow-up are shown in Figure 2 and discussed later.

The proportion of ‘yes’ responses to the diabetes items at baseline ranged from 57.1 to 70.4%. For COPD and CHD, the proportion of ‘yes’ responses at baseline was considerably lower; ranging from 16.1 to 42.9% for COPD and 5.4 to 26.8% for CHD, indicating that knowledge and understanding of these conditions were particularly limited at the start of the workshop. At the end of the two-day workshop, the proportion of ‘yes’ responses for all 10 condition-specific items increased significantly (*P*<0.0001), with most reaching 100% (range from 94.6 to 100).

Scores for the three items assessing relationships between mental and physical health and the role of psychological therapies in supporting people with long-term conditions were higher at baseline than for the condition-specific items, with ‘yes’ responses ranging from 76.8 to 94.6%. This proportion increased to 100% for all three items at the end of the workshop. The change was statistically significant for two of the items: ‘I understand how poor psychological wellbeing can impact physical health in people with long term conditions’ (*P*<0.05) and ‘I understand the role of psychological therapies in supporting people with long-term physical health conditions’ (*P*<0.0001). The only statement that did not demonstrate significant change from baseline was ‘I understand how living with a long-term physical health condition can impact on psychological wellbeing’. This is most likely due to ceiling effects (ie, scores at baseline close to 100%).

**Figure 3:**
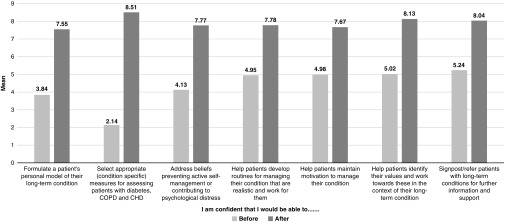
Self-efficacy ratings at baseline and follow-up

Mean scores for the self-efficacy items at baseline and follow-up are shown in Figure 3. Scores at baseline ranged from 2.14 (‘I am confident that I would be able to select appropriate (condition-specific) measures for assessing patients with diabetes, COPD and CHD’) to 5.24 (‘I am confident that I would be able to signpost/refer patients with long-term conditions for further information and support’). Scores for all seven items increased at the end of the workshop, ranging from 7.55 to 8.51.

Since self-efficacy items formed a reliable scale (Cronbach’s alpha=0.92 at baseline and 0.90 at follow-up), a total self-efficacy score was calculated summing across all seven items. The total score increased significantly from baseline (*M*=25.75; SD=13.41) to follow-up (*M*=55.45; SD=8.35). A paired-sample *t*-test revealed that this change was statistically significant (*P*<0.001).

### Open-ended comments

Participants reported that they found the workshop style to be informative and interactive and that they particularly valued the group activities, videos of patients with LTCs and training on outcome measures, as well as the resources provided for further information and support. Participants reported that they would value further training covering other LTCs (such as chronic pain, arthritis and medically unexplained symptoms), including information on other assessment and intervention approaches (particularly mindfulness-based therapies).

### Online training

The online training programme was accessed by 894 people in the first six months. Of these, 756 provided information on their occupation, including psychological wellbeing practitioners (*n*=270), counsellors (*n*=109), psychologists (*n*=56), other high-intensity therapists (*n*=264) and service managers/leads (*n*=57). Participants were employed within a wide range of NHS, voluntary and community organisations across the UK.

Feedback questionnaires were completed by 187 respondents. Responses are summarised in Table 1.

**Table 1:** Responses to online training feedback questionnaire

	Did you find the training?		
*n*(%)	Useful	Interesting	Easy to understand
Yes	165 (88%)	171 (91%)	180 (97%)
To some extent	21 (11%)	15 (8%)	6 (3%)
No	1 (1%)	1 (1%)	0
Total no. of respondents	187	187	186

The vast majority of respondents indicated that the training programme was useful, interesting and easy to understand. The mean satisfaction rating was 7.79 (median=8; mode=8, SD=2.51). Responses to the online training programme were consistent with feedback on the workshops, highlighting the importance of clearly presented information, interactive elements and links to sources of further information and support. Respondents also valued receiving feedback on their learning via the knowledge check questions. Suggestions for improvement included adding more video case studies, pictures and diagrams and including exam style questions, with a pass/fail rate.

## Discussion

Primary care mental health services are playing an increasingly important role in supporting people with co-morbid mental and physical health conditions. It is essential that staff employed in these services are able to develop an understanding of what it means to live with and manage a long-term condition on a day-to-day basis, as well as knowledge of relationships between mental and physical health and the role of psychological therapies in supporting people with LTCs. The aim of this project was to develop, deliver and evaluate a brief LTCs training workshop for primary care mental health staff in NW London. We also developed an online version of the training programme to be made available to mental health staff across the NHS.

Mental health staff participating in the workshop reported limited knowledge, understanding and confidence at baseline, underlining the need for training. For example, almost a third did not know the difference between Type 1 and Type 2 diabetes and a similar proportion indicated that they were unfamiliar with the symptoms of diabetes, did not know how diabetes is managed and did not understand the role of the patient in self-managing diabetes. Participants reported even lower levels of familiarity with COPD and CHD with only 16.1 and 5.4%, respectively, reporting awareness of the management of these conditions. Knowledge and understanding improved significantly following training; for most knowledge items, 100% of participants gave ‘yes’ responses after the workshop and all items were rated ‘yes’ by at least 96.4% of respondents. This increase in knowledge and understanding is notable, given that training was delivered over only two days. Self-efficacy also improved significantly following training. Hence, findings indicate that training provided the necessary conditions to not only improve participants’ knowledge of long-term conditions but also enhance their confidence in working effectively with this population.

Although the findings are encouraging, we acknowledge that changes in knowledge, understanding and confidence were examined immediately after training; hence, we do not know to what extent these changes will be maintained in the longer term. Also, we were unable to examine to what extent training resulted in changes in either clinical practice, or underlying clinical skills and competence. Future work will be needed to address these questions and to consider the implications for patient outcomes, including ‘soft’ outcomes, such as satisfaction with therapy and ‘hard’ outcomes, such as change in clinical measures of depression, anxiety and condition-specific distress. It will also be important to determine whether provision of psychological support benefits patient self-management and physical health outcomes. Future work may benefit from the use of validated outcome measures to assess changes in clinical competence, such as the Behaviour Change Counselling Index (Lane et al., 2005), which has been used to evaluate training for clinicians supporting people living with psoriasis (Chisholm *et al*., 2016).

The online training programme was accessed by almost 900 mental health staff during the first six months, with high levels of user satisfaction. Although online training platforms provide an opportunity to deliver training to larger numbers of people at minimal cost, some of the advantages of face-to-face training (eg, opportunities for group discussion, and ‘hands on’ activities) are more difficult to provide in this format. Nonetheless, our findings indicate that most people found this approach useful, interesting and easy to understand. Hence, online training could provide a useful adjunct to face-to-face training, particularly when new ways of working (and requisite knowledge, understanding and confidence) need to be disseminated across the workforce. Future work could examine the relative advantages/disadvantages of face-to-face versus online training in greater detail, including qualitative data on participant experience and quantitative data on clinician and patient outcomes.

Although the current project focussed specifically on primary care mental health staff, it is possible that other primary care staff, such as practice nurses and primary care physicians would find training on psychological aspects of managing LTCs useful. It is also possible that training could be extended to health professionals supporting patients with severe mental illness. Hence, a further avenue for future research would be to examine the training needs of other groups of health care staff involved in providing care and support to people with co-morbid mental and physical health conditions and to develop and evaluate training to meet these needs.

## Conclusions

Our findings indicate that mental health workers’ knowledge, understanding and confidence in providing effective support to patients with LTCs can be significantly improved via a brief training programme underpinned by psychology theory. Future work should examine impacts of training on clinician and patient outcomes, consider when/to what extent training may be delivered using online platforms and explore the potential benefits of extending training to other health care professionals.

## Financial Support

This work was supported by funding from Health Education England, North West London.

## Conflicts of Interest

None.

## Footnotes

1
Latest IAPT LTC guidance is available here: https://www.england.nhs.uk/wp-content/uploads/2018/03/improving-access-to-psychological-therapies-long-term-conditions-pathway.pdf

2
The IAPT LTC competence framework is available here: https://www.ucl.ac.uk/pals/research/cehp/research-groups/core/competence-frameworks/Psychological_Interventions_with_People_with_Persistent_Physical_Health_Problems
